# Social stigma and mental health

**DOI:** 10.1192/j.eurpsy.2021.978

**Published:** 2021-08-13

**Authors:** M. ValverDe Barea, C. Mata Castro, G.M. Ruiz Martinez, M.O. Solis

**Affiliations:** Unit Mental Health, Complejo Hospitalario Jaén, Jaén, Spain

**Keywords:** Stigma, Mental illness, mental health, community psychiatry

## Abstract

**Introduction:**

Stigma has been associated with various groups, based on certain attributes or characteristics, such as; Race or health status is a complex and dynamic process, a universal phenomenon that is part of all social groups and is maintained by its functions related to the establishment of one’s own identity and the facilitation of socialization processes. Many societies throughout history have identified people with a mental health problem as part of a minority group considered inferior to the rest. What has made this population an object of social stigma. With the beginning of community psychiatry, and with the need to integrate people with a serious mental disorder into it, it becomes even more valuable to be able to assess the social stigma towards mental illness in the community.

**Objectives:**

The goal is to examine community attitudes towards people with mental illness.

**Methods:**

Cross-sectional study of 228 people through an anonymous online survey. Sociodemographic variables and questionnaires were collected, such as the Community Attitudes Questionnaire towards people with Mental Illness (CAMI).

**Results:**

65% of respondents are women and 35% men. 74% have university studies. 18% do not agree that mental illness is an illness like any other. 1% believe that not all people can develop a mental illness. 7% of those surveyed are afraid that people with mental illness reside in their neighborhood and 14% believe that they are more dangerous people than the general population.

**Conclusions:**

Given the results obtained, we observe that the stigma towards people with mental illness is still present in society.

